# Supplementary material for: Dynamics of Ebola epidemics in West Africa 2014
Source: F1000Res. 2015 May 26;3:319. Originally published 2014 Dec 31. [Version 2] doi: 10.12688/f1000research.5941.2 (PMC4722695; doi:10.12688/f1000research.5941.2)
Supplement: Peer review history

# Peer review: Referee response for version 2

**DOI:** 10.5256/f1000research.7037.r8759

**Published:** 2015-05-27

**Authors:** Yang Kuang

**Affiliations:** 1 School of Mathematical and Statistical Sciences, Arizona State University, Tempe, AZ, USA

The authors adequately responded to my comment on the assumption that the transmission is constant.

I have read this submission. I believe that I have an appropriate level of expertise to confirm that it is of an acceptable scientific standard.

# Peer review: Referee response for version 1

**DOI:** 10.5256/f1000research.6350.r8331

**Published:** 2015-05-01

**Authors:** Yang Kuang

**Affiliations:** 1 School of Mathematical and Statistical Sciences, Arizona State University, Tempe, AZ, USA

This manuscript introduced some novel ways to connect the current West Africa Ebola outbreak to the standard SI model. However, the author routinely assumed that the infection rate β is a constant which more or less causes the exponential growth profile even when indications suggest otherwise after the month of October, 2014. The key to better modeling any infectious disease and especially in severe ones like Ebola, is to appropriately model the infection rate which is really a function of individual behavior. Arguably, individual behavior is often strongly correlated to the total cases reported. I suggest that the authors take a look at the paper by Chowell *et al.* below which implicitly incorporated such observations. One can show by a direct differentiation of the logistic model in the paper by Chowell *et al*. to find out that I'/I is assumed to be a linearly decreasing function of the total case reported.

Chowell G, Simonsen L, Viboud C, Kuang Y. 2014. Is West Africa approaching a catastrophic

phase or is the 2014 Ebola epidemic slowing down? Different models yield different answers for Liberia. PLOS Currents Outbreaks. 2014 Nov 20. Edition 1. doi: 10.1371/currents.outbreaks. b4690859d91684da963dc40e00f3da81. PLOS Currents Outbreaks.

I have read this submission. I believe that I have an appropriate level of expertise to confirm that it is of an acceptable scientific standard, however I have significant reservations, as outlined above.

# Response

**Published:** 2015-05-13

**Authors:** Musa Mammadov

**Affiliations:** Federation University, Australia

We thank the reviewer for pointing out the importance of the infection rate which is really a key focus of our paper. Our model mainly depends on two parameters:  β  and τ _2_ – time to isolation. Initially we also thought that β should be time dependent (in fact piece-wise constant) and obtained good data fitting in this way. However, later we observed that almost the same quality of data fitting can be achieved if we take β constant and vary only τ _2_. This is one the major findings of our paper and has important implications. For example:

- Since τ _2_ is an integer it is possible to generate future scenarios for the prediction of infectious population.
- As τ _2_ is time to isolation it can be linked with time to hospitalization and can lead to optimal control problems.

Such an optimal control model has already been implemented in our new study ( arXiv:1505.00872) where the problem of optimal distribution of bed capacities is investigated.

The paper by “Chowell *et al”* is cited in a new version regarding the behavior of β, alongside “Lekone *et al”* where β is considered as a control variable with an exponential decrease.

# Peer review: Referee response for version 1

**DOI:** 10.5256/f1000research.6350.r7898

**Published:** 2015-03-12

**Authors:** Gerhard-Wilhelm Weber

**Affiliations:** 1 Institute of Applied Mathematics, Middle East Technical University, Ankara, Turkey

This is an excellent research work for which the researcher(s) related to the study deserve great thanks and a particular recognition!

The paper discloses beauty and rigor of modern Mathematics and Operational Research, and it has the potential to strongly contribute to health and entire living conditions of individuals, communities and nations.

Moreover, it can very much stimulate and initiate future research works and practical contributions, e.g., via stochastic aspects and regime switches or paradigm changes.

I have read this submission. I believe that I have an appropriate level of expertise to confirm that it is of an acceptable scientific standard.
